# Targeted Disruption of Core 1 β1,3-galactosyltransferase (C1galt1) Induces Apical Endocytic Trafficking in Human Corneal Keratinocytes

**DOI:** 10.1371/journal.pone.0036628

**Published:** 2012-05-04

**Authors:** Ana Guzman-Aranguez, Ashley M. Woodward, Jesús Pintor, Pablo Argüeso

**Affiliations:** 1 Departamento de Bioquímica y Biología Molecular, Escuela Universitaria de Óptica, Universidad Complutense de Madrid, Madrid, Spain; 2 Schepens Eye Research Institute, Massachusetts Eye and Ear, Department of Ophthalmology, Harvard Medical School, Boston, Massachusetts, United States of America; Wayne State University, United States of America

## Abstract

**Background:**

Exposed mucosal surfaces limit constitutive endocytosis under physiological conditions to prevent uptake of macromolecules and pathogens and, therefore, cellular damage. It is now accepted that cell surface mucins, a group of high molecular weight glycoproteins on the epithelial glycocalyx, defined by their extensive O-glycosylation, play a major role in maintaining barrier function in these surfaces, but the precise mechanisms are unclear.

**Methodology/Principal Findings:**

In this work, we utilized a stable tetracycline-inducible RNA interfering system targeting the core 1 ß1,3-galactosyltransferase (C1galt1 or T-synthase), a critical galactosyltransferase required for the synthesis of core 1 O-glycans, to explore the role of mucin-type carbohydrates in apical endocytic trafficking in human corneal keratinocytes. Using cell surface biotinylation and subcellular fractionation, we found increased accumulation of plasma membrane protein in endosomes after C1galt1 depletion. Confocal laser scanning microscopy and fluorometry revealed increased translocation of negatively charged fluorescent nanospheres after C1galt1 knockdown sustained by an active transport process and largely independent of apical intercellular junctions. Internalization of nanospheres could be blocked by dynasore, nocodazole, chlorpromazine, and hyperosmotic sucrose, suggesting a mechanism for clathrin-coated pit budding and vesicular trafficking. This possibility was supported by experiments showing nanosphere colocalization with clathrin heavy chain in the cytoplasm.

**Conclusions/Significance:**

Together, the data suggest that core 1 O-glycans contribute to maintenance of apical barrier function on exposed mucosal surfaces by preventing clathrin-mediated endocytosis.

## Introduction

Rapid endocytosis and recycling of plasma membrane in mammalian cells contribute to the internalization of important nutrients, effector molecules (e.g., growth factors, hormones, antibodies), and macromolecules or particles that either specifically or non specifically bind to the cell surface [1]. Yet, endocytic activity can also be detrimental to cells, as protein toxins and microorganisms can exploit the cellular endocytic machinery to attack a given host. For many years, it has been recognized that exposed mucosal epithelia directly challenged by a microbe-rich environment are resistant to engulfment of noxious substances and infective particles located on their apical surfaces [2], [3]. In the eye, endocytic activity in corneal keratinocytes leads to internalization of viruses and bacteria [4], [5] and, therefore, to infection, a leading cause of blindness worldwide. This resistance to apical internalization also impairs the delivery of therapeutic components into the eye, as it is generally accepted that topical administration of macromolecules, such as genes or nanoparticles sized between 1 and 100 nanometers, cannot effectively cross the corneal epithelium barrier [6], [7]. To date, however, the molecular mechanisms limiting apical plasma membrane plasticity in these exposed mucosal surfaces remains largely unexplored.

The thick coat of glycans and glycoconjugates on the glycocalyx that emerges from apical membranes of epithelial cells is critical to preventing access and uptake of macromolecules and pathogens by mucosal surfaces. This glycocalyx is rich in cell membrane-associated mucins, a group of high molecular weight and heavily O-glycosylated glycoproteins with a long filamentous and anti-adhesive structure [8], [9], [10]. These tethered mucins restrict adenoviral and bacterial infection on mucosal surfaces [11], [12], [13], [14]. Integral to the function of cell surface mucins are their O-glycans and the Golgi-resident enzymes that synthesize them. The core 1 ß1,3-galactosyltransferase (C1galt1 or T-synthase) is a key enzyme that adds a galactose residue from the donor UDP-Gal to mucins and other glycoproteins containing GalNAcα1-Ser/Thr on mucin-type sequences to generate the core 1 disaccharide Galß1–3GalNAcα1-Ser/Thr or T-antigen [15]. The relationship between C1galt1 and mucin O-glycosylation has been demonstrated after genetic ablation of C1galt1 in mouse intestinal epithelium, showing that mucins purified from colon mucus lack core 1-derived O-glycans [16]. These mice also have impaired barrier function, as demonstrated by increased permeability to low molecular weight fluorescein isothiocyanate-dextran and the development of spontaneous colitis [16]. Similarly, data from mice deficient in glycosyltranferases responsible for the elongation of other O-glycan structures, such as core 2 β1,6-N-acetylglucosaminyltransferase (mucin-type) and core 3 β1,3-N-acetylglucosaminyltransferase, also indicate that mucin carbohydrates are required to maintain intestinal mucosal barrier function [17], [18]. While it has been proposed that altered O-glycosylation may impair the integrity of the mucus gel, a viscoelastic layer composed primarily of gel-forming mucins, allowing abnormal interaction between the extracellular milieu and the epithelium, little is known about the biologic mechanisms linking cell surface O-glycan abnormalities with altered permeability of the epithelial plasma membrane.

We recently developed a stable tetracycline-inducible RNA interfering system targeting C1galt1 to show that O-glycans on cell surface mucins contribute to the maintenance of apical barrier function in human corneal keratinocytes by interacting with the ß-galactoside-binding protein galectin-3 [19]. Multivalent galectin-3 is known to cross-link its counter-receptors, resulting in galectin-ligand lattices on the cell surface that are robust and resistant to lateral movement of membrane components [20]. In this study, we used the RNA interference system targeting C1galt1 to better understand the contribution of O-glycans to the regulation of endocytic trafficking in human corneal keratinocytes. Here, we show that targeted disruption of C1galt1 stimulates the endocytosis of plasma membrane proteins and enhances the internalization of nanoparticles in a clathrin-dependent manner.

## Results

**Figure 1:**
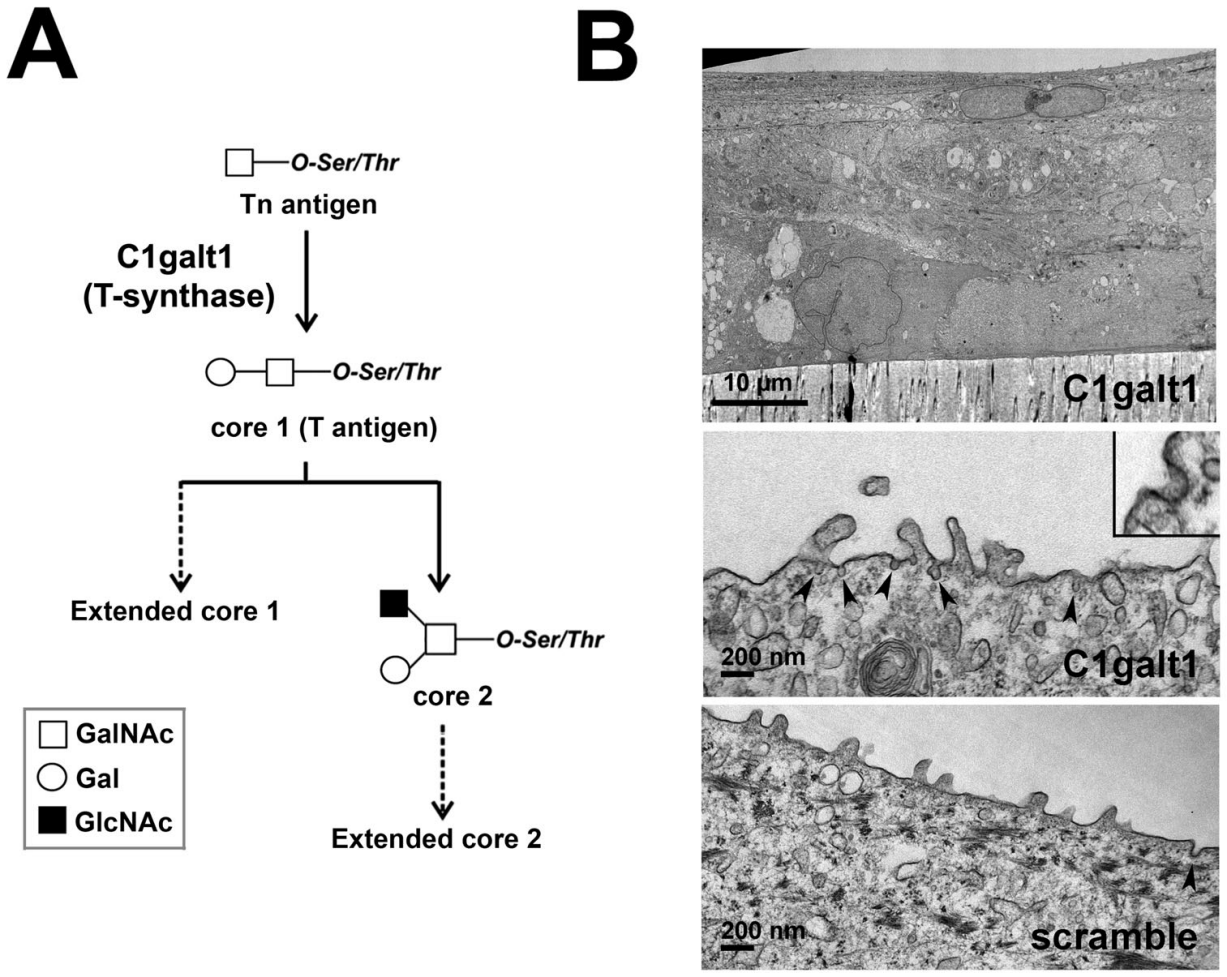
C1galt1-deficient human corneal keratinocytes display plasma membrane invaginations on the apical surface. (A) Scheme for mucin-type O-glycan biosynthesis. The core 1 β,3-galactosyltransferase (C1galt1 or T-synthase) is a key branchpoint enzyme that directs the synthesis of core 1 (T-antigen), the precursor structure for many extended mucin-type O-glycans in a wide variety of glycoproteins. (B) Electron micrograph at low magnification of an ultrathin section (60 to 90-Å) of C1galt1 keratinocytes grown on Transwell® inserts demonstrating cell stratification and the presence of flattened epithelial cells on the apical layer (top) Scale bar, 10 µm. Membrane invaginations (arrowheads, inset) can be observed occasionally on the apical portion of the plasma membrane in apical cells of C1galt1 keratinocytes (middle), more frequently than in scramble control (bottom). Scale bar, 200 nm.

A critical step in the biosynthesis of O-linked glycoproteins involves the synthesis of the core 1 disaccharide, also known as the T-antigen. Core 1 is the most common core structure found in mucins, and it serves as a key precursor for all core 1 and core 2 mucin-type O-glycans (Figure 1A). Results from our previous studies indicated that tetracycline-inducible human corneal keratinocytes stably transfected with C1galt1 shRNA are characterized by reduced core 1 and galectin-3 on the cell surface, concomitant with impaired barrier function [19]. To obtain insight into the plasma membrane morphology of C1galt1 shRNA keratinocytes, we examined epithelial cells cultured to stratification in Transwell® inserts by transmission electron microscopy. After induction of stratification by culture in serum-containing media for 7 days, membrane invaginations—typically with diameters of approximately 100 nm—were observed sporadically on the apical membrane of apical epithelial cells (Figure 1B), being more frequent (approx. 2-fold) in cells expressing C1galt1 shRNA than in scramble shRNA control. To determine whether targeted disruption of C1galt1 promotes endocytosis, we quantified the internalization of plasma membrane proteins using biotinylation and subcellular fractionation techniques.

### Abrogation of C1galt1 promotes membrane protein internalization

**Figure 2:**
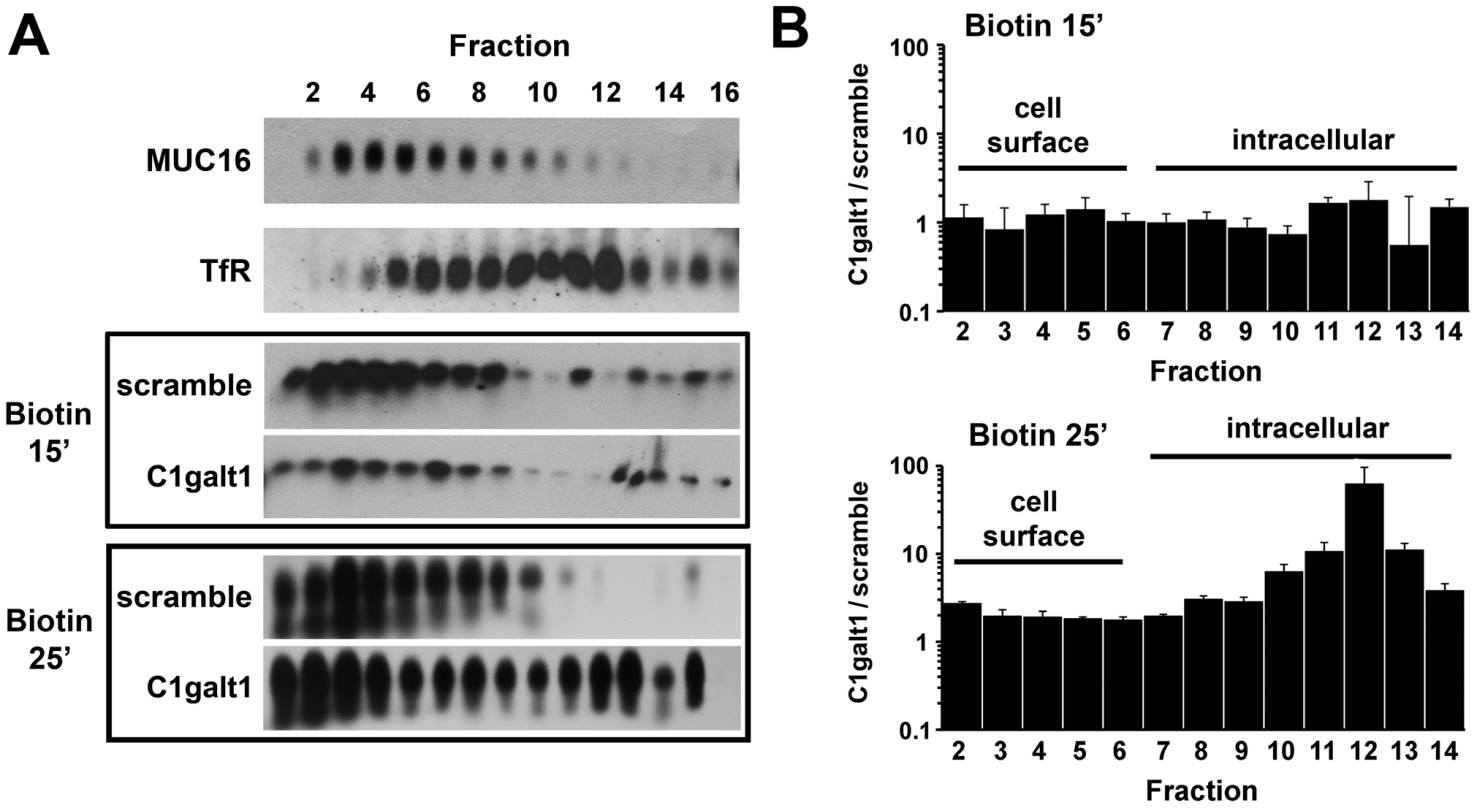
Targeted disruption of C1galt1 enhances endocytosis of biotinylated cell surface protein in corneal keratinocytes. (A) Cell surface protein was labeled with biotin at 4°C and then allowed to internalize for 15 or 25 min at 37°C. Crude postnuclear supernatants were ultracentrifuged using 5–25% Optiprep gradients and analyzed by agarose gel electrophoresis. Fraction 1 contains the lightest membranes; fraction 16 contains the densest membranes. The position of the plasma membrane and endocytic vesicles in the gradient was determined by western blot using antibodies to MUC16 and human TfR, respectively. Biotinylated protein was detected using streptavidin-peroxidase as described in Materials and Methods. (B) Quantitative evaluation of biotin accumulation in Optiprep density gradient. The graph shows the ratio of biotinylated protein in C1galt1 shRNA and scramble shRNA cells as determined by densitometry. Band intensity in each fraction was normalized to percent total biotin loaded in each gradient. Data for each condition are reported as the mean of three independent experiments.

Keratinocyte membrane proteins were biotinylated at 4°C and allowed to internalize at 37°C for 15 or 25 min. Postnuclear supernatants were then fractionated by Optiprep density gradient ultracentrifugation, and the endocytosis of biotinylated membrane protein was quantified by western blot using streptavidin-peroxidase staining. The position of the plasma membrane and endocytic vesicles on the gradient was determined in human corneal limbal epithelium-non transfected (HCLE-nt) control keratinocytes using antibodies to the membrane-associated MUC16 mucin and the human transferrin receptor (TfR), respectively. As shown in Figure 2, OptiPrep gradient separation revealed that abrogation of C1galt1 induces internalization of biotinylated protein in a time-dependent manner. At 15 min, most biotinylated protein in scramble shRNA and C1galt1 shRNA cells sedimented in low-density regions of the gradient (fractions 2–6), corresponding to the plasma membrane region. Internalization of cell surface protein was observed, however, in C1galt1 shRNA cells following incubation with biotin for 25 min. At this time point, biotinylated protein was found in high-density fractions of the gradient (fractions 5–12), corresponding to endosomes. Thus, these results provide evidence that disruption of core 1 O-glycosyltransferase activity induces endocytosis and faster accumulation of cell surface proteins in cytoplasmic vesicles.

### Core 1 O-glycosylation is required to maintain transcellular barrier function

**Figure 3:**
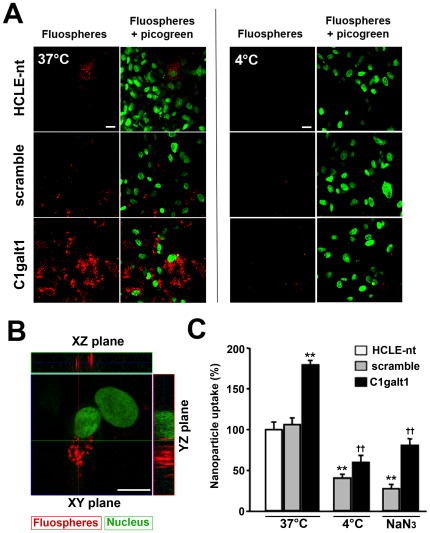
Targeted disruption of C1galt1 enhances nanoparticle uptake in human corneal keratinocytes. (A) HCLE-nt, scramble shRNA, and C1galt1 shRNA keratinocytes were incubated from the apical side with 100 nm FluoSpheres® suspensions (10^10^ nanoparticles/ml) for 3 h at 37°C, and subsequently examined by confocal microscopy. The position of cell nuclei was determined with PicoGreen. An increased number of nanospheres was observed in C1galt1 shRNA cells as compared to controls. When experiments were carried out at 4°C, a condition that blocks active transport processes, the internalization of nanospheres was dramatically reduced. Scale bar, 20 µm. (B) Examination of Z-stacked images showed that the nanospheres localized predominantly to the cytoplasm in C1galt1 shRNA cells incubated at 37°C. Scale bar, 20 µm. (C) Quantitative analyses of nanoparticle internalization were carried out by fluorometry of cell lysates. Nanoparticle internalization was significantly higher in C1galt1 cells as compared to controls. The uptake was dependent on the temperature and was impaired by treatment with the metabolic inhibitor sodium azide. Values are normalized to percent total uptake in HCLE-nt cells. Data for each condition are reported as the mean of three wells in three independent experiments. **P<0.001 compared with scramble shRNA group at 37°C and ††*P*<0.001 compared with C1galt1 shRNA group at 37°C.

Maintenance of an effective epithelial barrier requires both trans- and paracellular exclusion of macromolecules and pathogenic particles to prevent damage to exposed mucosal surfaces. Because of our observation that C1galt1 cells have increased apical endocytic activity, we sought to determine whether abrogation of C1galt1 would enhance the internalization of apically applied particles. For these experiments, we used fluorescent polystyrene nanoparticles (FluoSpheres®), which have been employed as a model system to mimic the interaction between host cells and pathogenic organisms in the absence of microbial molecular signaling and to study mucosal permeability [12], [21], [22]. Figure 3A shows confocal images of 100-nm-diameter, negatively charged, fluorescent nanoparticles apically applied onto corneal keratinocytes. A robust intracellular accumulation of nanoparticles was observed in C1galt1 shRNA keratinocytes at 37°C. This increase was not observed at 4°C, when active transport processes were blocked, indicating that abrogation of core 1 glycosyltransferase activity enhances particle internalization in an energy-dependent manner. Control cells, both non-transfected (HCLE-nt) and scramble shRNA-transfected, showed some degree of particle internalization, probably due to incomplete differentiation of some apical cells in our *in vitro* model of stratification [23]. The intracellular location of nanoparticles in C1galt1 shRNA cells was confirmed in orthogonal confocal images (Figure 3B).

Internalization of Fluospheres® after C1galt1 abrogation was further quantified by fluorometry. As shown in Figure 3C, the uptake of nanoparticles by C1galt1 shRNA cells increased significantly, by 1.7-fold relative to HCLE-nt and scramble control keratinocytes. To confirm the mechanism by which these particles entered the cells, we repeated these internalization experiments at 4°C and in the presence of the metabolic inhibitor sodium azide to inhibit active transport processes. Under these conditions, the internalization of nanoparticles by the cells was consistently reduced by more than 2-fold in both scramble and C1galt1 shRNA cells, corroborating our confocal microscopy findings, and confirming that nanoparticle uptake in corneal keratinocytes is an active endocytic process.

**Figure 4:**
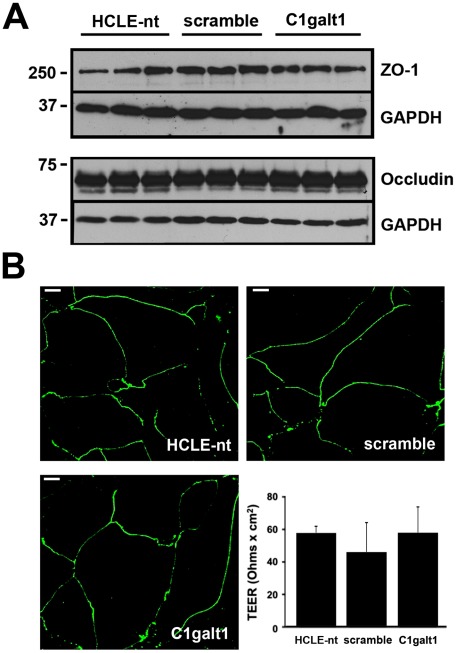
C1galt1 abrogation does not alter tight junction integrity or function. (A) Whole-cell protein lysates (15 µg) were resolved on SDS-PAGE and immunoblotted with antibodies against ZO-1, occludin and GAPDH, the latter serving as loading control. Comparable levels of junctional proteins were observed in C1galt1 shRNA keratinocytes as compared to controls. (B) Stratified cultures of corneal keratinocytes were fixed, permeabilized and stained for ZO-1. Confocal images corresponding to horizontal sections demonstrated ZO-1 distribution to the cell border in all cultures analyzed, consistent with tight junction localization in normal corneal epithelial cells [39]. Permeability measurements showed that TEER responses were not attenuated in C1galt1 shRNA cells as compared to controls. Data for each condition are reported as the mean of three wells in three independent experiments. Scale bar, 20 µm.

In addition to the epithelial glycocalyx, a second line of defense in mucosal surfaces is formed by intercellular junctions that seal the paracellular space and connect individual epithelial cell membranes [24]. To exclude the possibility that abrogation of C1galt1 altered the paracellular transport of FluoSpheres®, we examined the integrity and distribution of tight junction proteins, as well as transepithelial electrical resistance (TEER), a measure of tight junction permeability across cultures of stratified keratinocytes. By western blot, similar levels of the junction proteins ZO-1 and occludin were observed in HCLE-nt, scramble, and C1galt1 shRNA cells (Figure 4A). ZO-1 in the cell cultures localized primarily to junctional areas or cell peripheries (Figure 4B). This is consistent with the presence of a functional intercellular barrier. Finally, analyses by TEER in corneal keratinocytes grown on Transwell® inserts further confirmed that abrogation of C1galt1 did not significantly impair paracellular permeability (Figure 4B, insert). Together, these experiments indicate that transcellular transport, and not paracellular transport, is the major pathway for nanoparticle internalization in corneal keratinocytes following abrogation of core 1 O-glycosylation.

### Disruption of C1galt1 in corneal keratinocytes induces clathrin-mediated endocytosis

**Figure 5:**
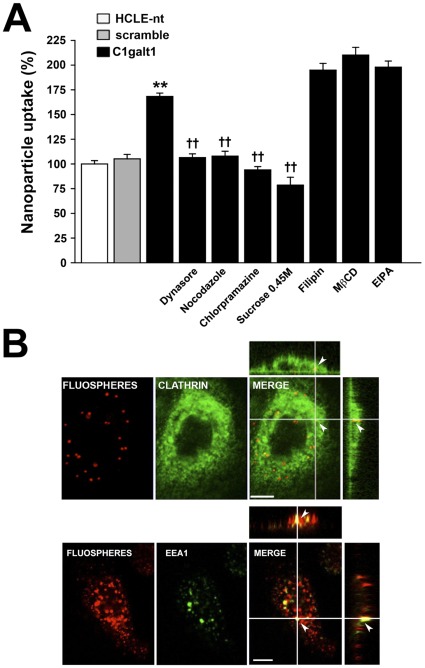
Clathrin-mediated endocytosis is a predominant pathway for nanoparticle internalization in C1galt1 shRNA cells. (A) For fluorometry assays, corneal keratinocytes were pre-incubated at 37°C in the presence of inhibitors of endocytosis. After 30 min, FluoSpheres® suspensions (10^10^ nanoparticles/ml) were added to the cells in the continuous presence of inhibitors and incubated for 3 h at 37°C. Sucrose was directly added to the suspension in these experiments. Nanoparticle uptake was quantified by fluorometry as described in Materials and Methods. Values are normalized to percent total uptake in HCLE-nt cells. Data for each condition are reported as the mean of three wells in three independent experiments. **P<0.001 compared with scramble shRNA group and ††*P*<0.001 compared with the C1galt1 shRNA group with no inhibitors. (B) Confocal microscope images of C1galt1 shRNA cells. For colocalization experiments, keratinocytes were incubated with FluoSpheres®suspensions (red) for 3 h at 37°C. The cells were then fixed, permeabilized and stained for clathrin heavy chain or EEA1 (green). Examination of Z-stacked images shows colocalization of nanospheres with clathrin (Pearson's coefficient: 0.939) or EEA1 (Pearson's coefficient: 0.885) in the cytoplasm (arrowheads). Scale bar, 10 µm.

Endocytosis occurs by multiple mechanisms that fall into two broad categories, (1) phagocytosis, typically restricted to specialized mammalian cells, and (2) pinocytosis, which occurs in all cells by at least four basic mechanisms: macropinocytosis, clathrin-mediated endocytosis, caveolae-mediated endocytosis, and clathrin- and caveolae-independent endocytosis [25]. In the next series of experiments, to determine the mechanism of enhanced internalization of FluoSpheres® after C1galt1 abrogation, the uptake of nanoparticles was evaluated under conditions known to inhibit specific endocytic pathways. As shown in Figure 5A, treatment of C1galt1 shRNA keratinocytes with dynasore, an inhibitor of the guanosine triphosphatase activity of dynamin (essential for clathrin-dependent coated vesicle formation), and nocodazole, which induces microtubule depolymerization, both reduced the uptake of nanoparticles to levels observed in control cells, suggesting a mechanism for coated pit budding and vesicular trafficking in nanoparticle uptake. Moreover, chlorpromazine and hyperosmotic sucrose, which induce redistribution of the clathrin-coated pit component AP-2 from the plasma membrane to endosomes, and clathrin dissociation, respectively, also significantly inhibited nanoparticle uptake. By contrast, inhibitors of endocytic mechanisms involving caveolae (filipin), lipid raft formation (methyl-ß-cyclodextrine, MβCD) or macropinocytosis (5-(N-ethyl-N-isopropyl)amiloride, EIPA) did not influence nanoparticle internalization in C1galt1 shRNA cells.

Additional confocal microscopy experiments were performed to observe the distribution of nanoparticles in relation to clathrin and the early endosome marker EEA1 (Early Endosome Antigen 1). Scramble and C1galt1 shRNA keratinocytes were incubated with FluoSpheres® for 3 h at 37°C followed by immunohistochemistry with an anti-clathrin heavy chain or anti-EEA1 antibodies. As expected, the anti-clathrin antibody bound to numerous punctate loci found throughout the cytoplasm of the cell (Figure 5B). A subset of the clathrin was found to colocalize with the FluoSpheres® in scramble (data not shown) and C1galt1 shRNA cells. In these experiments, some nanoparticles did not display colocalization with clathrin. This was not totally unexpected, as it is possible to speculate that FluoSpheres® reach other intracellular compartments following disassembly of the coated vesicle [26]. This hypothesis was supported by experiments showing colocalization of a fraction of nanoparticles with EEA1 (Figure 5B). Altogether, these results indicate that clathrin-mediated endocytosis is a predominant mechanism for nanoparticle internalization in corneal keratinocytes lacking core 1 O-glycosylation.

## Discussion

The mechanisms that govern the exclusion of macromolecules and microorganisms from mucosal surfaces are of great importance for understanding infection and to enhance the delivery of therapeutic molecules into epithelial cells. It has long been recognized that an extracellular layer of secreted mucus provides an effective biophysical barrier that traps particulate material and pathogens [27], [28]. A second barrier on mucosal surfaces is composed of membrane-associated mucins on the epithelial glycocalyx that can extend up to 500 nm above the plasma membrane and, therefore, provide steric hindrance [12]. Mucin carbohydrates are central to the mucosal barrier, and their abrogation has been associated with increased mucosal permeability [16], [17], [18]. Here, we provide further evidence showing that O-glycans contribute to maintenance of barrier function on the apical surface by preventing clathrin-mediated endocytosis in human corneal keratinocytes.

We first demonstrated that targeted disruption of C1galt1, the only branching glycosyltransferase that can synthesize core 1 O-glycans on mucins and other O-linked glycoproteins (Figure 1A), promoted endocytosis of biotinylated cell surface protein. This result is consistent with previously published data showing that, in glycosylation-defective Chinese hamster ovary ldlD cells, underglycosylation of cell surface MUC1 mucin stimulates its endocytosis through clathrin-coated pits [29]. This process is modulated by the highly O-glycosylated ectodomain, since MUC1 chimeras lacking the extracellular domain are internalized faster than MUC1 in Chinese hamster ovary cells [30]. Conversely, inhibition of α2–3-sialylation in IMIM-PC-1 pancreatic cancer cells by the O-glycan sugar analog benzyl-α-GalNAc was associated with a slower rate of endocytosis but enhanced accumulation of apical glycoproteins in cytoplasmic vesicles (possibly due to defective recycling to the plasma membrane), and marked morphological changes, including increased cell size and accumulation of numerous electron-lucid cytoplasmic vesicles [31]. In our experiments using transmission electron microscopy, we did not observe any apparent alteration in cell morphology in C1galt1 shRNA cells as compared to scramble or HCLE-nt controls (data not shown), suggesting that the effect of O-glycan abrogation on endocytic trafficking and cell morphology is cell-type specific and may depend on the structural organization and interactions of individual carbohydrates on the glycocalyx.

Exposed mucosal surfaces limit constitutive endocytosis under physiological conditions to prevent uptake of toxins and pathogens and, therefore, avoid cellular damage. In these surfaces, as in most mammalian cells, membrane-bound internalization of macromolecules and microorganisms larger than a few nanometers in size require disruption of the integrity of the biological barriers, including the glycocalyx [32]. Despite the abundance of cell surface carbohydrates on epithelial glycocalyces, little is known about their roles, or the roles of the enzymes that synthesize them, in particle penetration. The fact that apical C1galt1 shRNA cells internalized cell surface-bound proteins more efficiently than control cells led us, in subsequent experiments, to determine whether disruption of core 1 O-glycan biosynthesis would enhance the uptake of extracellular nanoparticles. Our results revealed increased particle internalization in stratified cultures of C1galt1 shRNA cells (Figure 3A), supporting the role of mucin-type O-glycans in barrier formation. Internalization was energy dependent, as shown by incubation of cells at 4°C or pretreatment with the metabolic inhibitor sodium azide [33], [34], and involved the assembly of clathrin and accumulation of coated pits, as shown by use of dynasore, chlorpromazine, and hyperosmotic sucrose—known inhibitors of clathrin-coated pit assembly and progression [35], [36]. Moreover, a subset of nanoparticles colocalized and accumulated with clathrin and EEA1 in the cytoplasm, as demonstrated by immunofluorescence microscopy (Figure 5B), which further supports a role for clathrin-mediated endocytosis in nanoparticle internalization by human corneal keratinocytes with altered core 1 O-glycosylation.

In previous studies, we showed that cell surface mucins interact with galectin-3 in human corneal keratinocytes and that abrogation of C1galt1 reduces cell surface galectin-3, while increasing epithelial permeability to rose bengal diagnostic dye [19]. Galectin-3 is a multivalent, ß-galactoside-binding lectin implicated in the regulation of endocytosis through binding carbohydrate ligands on cell surface glycoprotein receptors [37]. Therefore, we now speculate that, when bound to cell surface O-glycans, galectin-3 effectively promotes lattice formation and prevents the endocytosis of plasma membrane proteins and extracellular material. This model is supported by previous data indicating that β1,6-N-acetylglucosaminyltransferase V (Mgat5) can modify N-glycans on EGF and TGF–β receptors to promote galectin-3 cross-linking at the cell surface and delay their removal by constitutive endocytosis [38].

Collectively, our results indicate that targeted disruption of C1galt1 in human corneal keratinocytes induces endocytosis of plasma membrane components and promotes internalization of extracellular macromolecules in a clathrin-dependent manner. While these data support a mechanism by which O-glycans on the epithelial glycocalyx limit constitutive endocytosis to promote barrier function, future experiments are needed to further characterize the role of galectin-3 interactions in preventing cellular damage to exposed mucosal surfaces. Moreover, these data also support the concept that transient manipulation of O-glycans in the glycocalyx is an alternative approach to delivering therapeutic nanoparticles into mucosal surfaces.

## Materials and Methods

### Cell culture

All work was done in human corneal keratinocytes stably transfected with tetracycline-inducible C1galt1 shRNA or scramble shRNA. Incubation of corneal keratinocytes after stable expression of C1galt1 shRNA with doxycycline results in decreased levels of C1galt1 mRNA and protein, and impaired cell surface mucin O-glycosylation as shown by staining with peanut agglutinin, a lectin that recognizes core 1 O-glycans, in biotinylation assays [19]. Human corneal keratinocytes expressing a tetracycline repressor, but not transfected with shRNA, were used as an additional control (HCLE-nt). The techniques used for the development of the cell lines have been previously described [19].

Cells were cultured in keratinocyte serum-free medium (Invitrogen, Carlsbad, CA) supplemented with 0.4 mM CaCl_2_, 25 µg/ml bovine pituitary extract, 0.2 ng/mL epidermal growth factor, 1% penicillin/streptomycin and 3 µg/ml blasticidin, and maintained at 37°C in 5% CO_2_. After reaching confluence, cell cultures were grown in Dulbecco's modified Eagle's medium/F12 (DMEM/F12; Cellgro, Herndon, VA) supplemented with 10% calf serum, 10 ng/ml EGF, 1% penicillin/streptomycin, and 3 µg/ml blasticidin for 7 days to promote stratification and differentiation, as previously reported [23]. Doxycycline, a synthetic derivative of tetracycline, was administered to cells for 4 days (nanoparticle uptake studies) or 7 days after reaching confluency to induce shRNA expression in transfected cells.

### Transmission electron microscopy

Stratified human corneal keratinocytes were cultured, as described above, in Transwell® cell culture inserts (Corning Inc., Corning, NY). After discarding the media, cell cultures were rapidly fixed in Karnovsky's fixative (2% paraformaldehyde, 2.5% glutaraldehyde in 0.1 M cacodylate buffer, pH 7.4). Cells were then post-fixed in 1% osmium tetraoxide and en bloc-stained with 0.5% uranyl acetate for 30 min. Cells were then dehydrated in graded ethanol solutions and propylene oxide before being infiltrated in EMbed 812. A diamond knife ultramicrotome (LKB Ultramicrotome, Bromma, Sweden) was used to cut transversely to the plane of the insert. Sixty- to 90-Å ultrathin sections were examined with a transmission electron microscope (model 410; Philips Electronics NV, Eindhoven, The Netherlands) equipped with a Gatan ES1000W Erlangshen CCD Camera for acquisition of digital images.

### Biotinylation and subcellular fractionation

Cell surface biotinylation of stratified human corneal keratinocytes was performed using the Pierce® Cell Surface Protein Isolation Kit (Thermo Fisher Scientific, Rockford, IL). Cells grown in T75 tissue culture flasks were labeled with sulfo-NHS-SS-biotin for 30 min at 4°C. Following quenching, cultures were immediately washed three times with phosphate-buffered saline (PBS) and incubated with supplemented DMEM/F12 pre-warmed at 37°C for 15 or 25 min. Cells were then scraped in homogenization buffer (0.25 M sucrose, 78 mM KCl, 4 mM MgCl_2_, 8.4 mM CaCl_2_, 10 mM EGTA, 50 mM Hepes-NaOH, pH 7.0) and lysed by 20 passages through a 20G needle and 20 passages through a 25G needle. Cellular debris and nuclei were pelleted by centrifugation at 1,000 *g* for 5 min. Plasma and intracellular membranes were fractionated on a continuous 5–25% Optiprep gradient (Axis-Shield, Dundee, Scotland) prepared by diffusion of discontinuous gradients according to the manufacturer's instructions. Postnuclear supernatants were overlaid to the top of 13 ml gradients and centrifuged at 90,000 *g* for 20 h at 4°C in a Sorvall TH-641 rotor. Fractions of 500 µl were collected from the top of the gradient. For analyses, proteins were precipitated by addition of deoxycholate/trichloroacetic acid, collected by centrifugation, and washed twice for 30 min in 80% acetone. Samples were resolved under reducing conditions by 1% agarose gel electrophoresis, and transferred onto nitrocellulose membranes (Bio-Rad, Hercules, CA) by vacuum blotting. Membranes were blocked overnight, then incubated with antibodies to MUC16 (M11; 1∶3000; NeoMarkers, Fremont, CA) and human TfR (H68.4; 1∶1000; Invitrogen). After incubation with the appropriate peroxidase-conjugated secondary antibodies (Santa Cruz Biotechnology, Santa Cruz, CA), positive binding was detected using the SuperSignal West Pico Chemiluminescent Substrate (Pierce, Rockford, IL). Biotinylated protein was detected on the membranes after blocking with Tris-buffered saline Tween (TBS-T) using a biotin-streptavidin-peroxidase kit (Vectastain® ABC Kit; Vector Laboratories, Burlingame, CA). Band intensities were quantified by densitometry using ImageJ software (National Institutes of Health, Bethesda, MD; http://rsb.info.nih.gov/nih-image).

### Nanoparticle uptake in corneal keratinocytes

One hundred nm carboxylate-modified red fluorescent nanoparticles (FluoSpheres®, Excitation 580 nm/Emission 605 nm; Molecular probes, Eugene, OR) were incubated for 1 h with 1% bovine serum albumin in an ultrasonic bath to reduce nanoparticle aggregation. Stratified human corneal keratinocytes grown on glass chamber slides (Nalgene Nunc International, Naperville, IL) were incubated with FluoSpheres® suspensions (10^10^ nanoparticles/ml) in serum-free DMEM/F12 for 3 h at 4°C or 37°C. After incubation, the suspensions were aspirated, and the cell cultures washed three times with ice-cold PBS to remove excess nanoparticles. Subsequently, cells were fixed for 15 min in 4% paraformaldehyde and further rinsed in PBS. To determine the position of the cell nuclei, cells were incubated with PicoGreen (Invitrogen) diluted 1∶200 in TE buffer (10 mM Tris, 1 mM EDTA, pH 7.5) for 15 min in the dark. After washing with TE buffer, samples were mounted with Vectashield Mounting Medium (Vector Laboratories) and examined with a Zeiss Axiovert 200 M confocal microscope equipped with a LSM 5 Pascal confocal module (Carl Zeiss Meditec GMhb, Jena, Germany).

Nanoparticle uptake was further quantified by fluorometry. Stratified keratinocytes grown on 12-well cell culture plates (Corning, Inc.) were incubated with FluoSpheres® suspensions as described above. After three washes with ice-cold PBS, the cells were solubilized in 0.1 M NaOH/5% SDS, and the cell lysates from each well used to quantify fluorescence using a Varioskan Flash spectral scanning multimode reader (Thermo Fisher Scientific). For inhibition studies, cells were pre-incubated with one of the following inhibitors for 30 min at 37°C: dynasore (5 µg/ml), nocodazole (5 µg/ml), chlorpromazine (10 µg/ml), filipin (5 µg/ml), MβCD (10 mM), or EIPA (25 µg/ml) (all from Sigma-Aldrich, St. Louis, MO). Thereafter, cells were incubated with FluoSpheres® suspensions for 3 h at 37°C in the presence of the inhibitors. Sucrose (0.45 M) and sodium azide (100 mM) were added directly to the nanoparticle suspension in these experiments. Inhibitor toxicity was evaluated in preliminary experiments (data not shown) and only concentrations maintaining cell viability were used. Dynasore is a specific endocytic inhibitor for dynamin with an IC_50_ of ∼15 µM (5 µg/ml). The specificity of the other inhibitors was validated using FITC-transferrin as model cargo (data not shown). Levels of nanoparticle uptake in fluorometry analyses are relative to HCLE-nt control. Data for each condition are reported as the mean of three wells in three independent experiments.

### Western blotting of junctional proteins

Total cellular protein was extracted using RIPA buffer (150 mM NaCl, 50 mM Tris, pH 8.0, 1% NP 40, 0.5% deoxycholate, 0.1% SDS) plus Complete Protease Inhibitor Cocktail (Roche Biochemical, Indianapolis, IN). Protein concentration was determined using the Pierce BCA Protein Assay Kit (Thermo Scientific). Samples were resolved under reducing conditions on 4% stacking and 10% separating SDS-PAGE gels, followed by transfer onto nitrocellulose membranes. Membranes were blocked with 5% non-fat dry milk, then incubated with antibodies to ZO-1 (Z-R1; 1∶500, Invitrogen) and occludin (OC-3F10; 1∶250, Invitrogen). Binding of GAPDH antibody (1∶2000, Santa Cruz Biotechnology) was used as loading control. After incubation with the appropriate peroxidase-conjugated secondary antibodies (Santa Cruz Biotechnology), positive binding was detected using the SuperSignal West Pico Chemiluminescent Substrate (Pierce).

### Immunofluorescence confocal microscopy

The tight junction protein ZO-1 was examined in stratified keratinocytes by immunofluorescence confocal microscopy. Cells on chamber slides were fixed in absolute methanol at −20°C for 15 min, washed with PBS and permeabilized with 0.02% Tween 20 in PBS at room temperature for 10 min. Cells were then blocked with 4% BSA in PBS for 10 min and incubated with ZO-1 antibody (ZMD.436; 1∶50; Invitrogen) overnight at 4°C. After washing with PBS, cells were incubated with FITC-conjugated secondary antibody. Incubation with primary antibodies was routinely omitted in control experiments. Samples were mounted with Vectashield Mounting Medium (Vector Laboratories) and examined using a confocal laser-scanning microscope as described above.

In addition, inmunofluorescence confocal experiments were performed to determine whether internalized nanospheres colocalized with clathrin and the early endosome marker EEA1. Stratified keratinocytes were incubated with fluorescent nanospheres as described above. After fixation in 4% paraformaldehyde, the cells were permeabilized with 0.25% Triton X-100 in PBS. Non-specific binding sites were blocked with PBS containing 1% BSA prior to incubation with a clathrin heavy chain antibody (E-11; 1∶50, Santa Cruz Biotechnology) or rabbit polyclonal anti-EEA1 (ab 2900; 1∶100, Abcam, Cambridge, MA) overnight at 4°C. After washing with PBS, cells were incubated with FITC-conjugated goat anti-mouse or anti-rabbit IgG (1∶100; Jackson ImmunoResearch Laboratories, West Grove, PA), respectively, for 1 h at room temperature. Cultures were examined by confocal microscopy as described above. Pearson's colocalization coefficients were calculated using the JACoP tool of ImageJ software.

### Transepithelial electrical resistance (TEER)

TEER was determined on stratified cells grown in Transwell® cell culture inserts with an Evom^2^ Epithelial Voltohmmeter (World Precision Instruments, Sarasota, FL). Before each measurement, the Evom^2^ was “zeroed” according to the manufacturer's instructions. One Transwell® insert was left empty as a control to determine the intrinsic resistance of the filter, which was subtracted from all readings. Experiments were performed in triplicate and the transepithelial resistance (ohm-square centimeters) was calculated by multiplying the measured electrical resistance by the area of the filter (1.12 cm^2^).

### Statistical analysis

Statistical comparisons of the data were performed using the one-way ANOVA test. All the statistical analyses were performed using InStat3 software (GraphPad Software, La Jolla, CA).
